# Enumerating and indexing many-body intramolecular interactions: a graph theoretic approach

**DOI:** 10.1007/s10910-015-0510-x

**Published:** 2015-05-19

**Authors:** Robert Penfold, Peter J. Wilde

**Affiliations:** Institute of Food Research, Norwich Research Park, Colney, Norwich, NR4 7UA UK

**Keywords:** Computer simulation, Molecular modelling, Graph theory, 82-08, 05A15, 94C15

## Abstract

The central idea observes a recursive mapping of $n$-body intramolecular interactions to $(n+1)$-body terms that is consistent with the molecular topology. Iterative application of the line graph transformation is identified as a natural and elegant tool to accomplish the recursion. The procedure readily generalizes to arbitrary $n$-body potentials. In particular, the method yields a complete characterization of $4$-body interactions. The hierarchical structure of atomic index lists for each interaction order $n$ is compactly expressed as a directed acyclic graph. A pseudo-code description of the generating algorithm is given. With suitable data structures (e.g., edge lists or adjacency matrices), automatic enumeration and indexing of $n$-body interactions can be implemented straightforwardly to handle large bio-molecular systems. Explicit examples are discussed, including a chemically relevant effective potential model of taurocholate bile salt.

## Introduction

The implementation of computer code for realistically simulating the configurations and motion of molecular objects requires modelling of many-body through-bond interactions. In turn, it is necessary to identify the participating atoms in each interaction. This paper presents a novel and exhaustive enumeration procedure exploiting the line graph transformation of the graph that encodes the molecular structure. In principle, by virtue of the recursive nature of the algorithm, straightforward extension to arbitrarily high order interactions is possible.

Application of graph theoretic methods to the general study of molecular structure, and to equilibrium statistical mechanics in particular, are not new. Significant examples include the development of molecular branching rules [1], the enumeration of isomers and the definition of topological indexes [2], as well as the analysis of discrete lattice models [3] and Mayer’s cluster decomposition of the $2$-body configuration integral [4].

Modelling and theory distinguish between simple materials, comprised of weakly interacting elementary units, and complex materials including objects with internal structure characterized by relatively strong coupling and typically mimicking the covalent architecture of molecular species [5]. Furthermore, intramolecular interactions can be treated either quantum mechanically (Car–Parrinello method [6] with density functional theory of electronic structure [7]) or by a classical effective potential obtained through some more or less ad hoc coarse-graining procedure [8, 9]. In molecular dynamics or equilibrium Monte Carlo simulations, for example, the total intramolecular potential energy is typically decomposed as [10]

1 $$\begin{aligned} \begin{aligned} \mathcal{U}_\mathrm{intra}\bigl ( \bigl \{ \mathbf{r}_{s} \bigr \} \bigr )&= \underbrace{ \sum _\mathrm{bonds} \mathcal{U}_{2}\bigl ( \mathbf{r}_{i},\ \mathbf{r}_{j} \bigr ) }_\mathrm{topology}\\&\quad + \underbrace{ \sum _\mathrm{bends} \mathcal{U}_{3}\bigl ( \mathbf{r}_{i},\ \mathbf{r}_{j},\ \mathbf{r}_{k} \bigr ) + \sum _\mathrm{torsions} \mathcal{U}_{4} \bigl ( \mathbf{r}_{i},\ \mathbf{r}_{j},\ \mathbf{r}_{k},\ \mathbf{r}_{l} \bigr ) + \cdots }_\mathrm{flexibility} \end{aligned}, \end{aligned}$$

where the sums range over suitable $n$-body potentials $\mathcal{U}_{n}$ that depend on the spatial configuration $\bigl \{\mathbf{r}_{s}\bigr \}$ of the constituent atoms. Similar expansions are developed for non-bonded forces too, and may also include $1$-body coupling to an external field, but the indiscriminate character of these through-space interactions usually means that the identification of participating atoms is determined by a simple range parameter. For intramolecular interactions, however, the enumeration and indexing of atoms involving in each sum of (1) is subject to the constraints of molecular topology. Indeed, the $2$-body bond interactions $\mathcal{U}_{2}$*define* the molecular framework, while the higher order terms in (1) serve to model the more or less restricted molecular flexibility associated with bond hybridization or electronic delocalisation (e.g., aromaticity and resonance structures). The selection of these higher order potentials is often based on chemical intuition and are typically supplied to simulation software as user defined input. Mature and widely used simulation packages (e.g., GROMACS, LAMMPS, NAMD, etc.) invariably support highly optimized force fields (e.g., CHARMM, AMBER, OPLS, MMFF, etc.) that faithfully represent detailed atomistic structures. An alternative approach is to input only the molecular topology, then systematically generate all possible many-body index lists from this information and invite the user to select non-zero force constants and appropriate functional forms for the required potentials. This paradigm is more natural for the implementation of coarse-grained models derived by thermodynamic considerations (e.g., MARTINI [11]). To facilitate this semi-automatic procedure, a graph theoretic construction is developed here to exhaustively enumerate and index arbitrary $n$-body intramolecular interactions starting from the description of $2$-body adjacency.

The central idea in this work observes the correspondence between the hierarchy of $n$-body intramolecular interactions and iteration of the line graph [12] transformation $L(G)$ on a connected “molecular” graph $G$.

## Graph theory

A simple, connected and undirected graph $G=\bigl (V(G),\ E(G)\bigr )$ is composed of a finite nonempty vertex set $V(G)=\bigl \{v_{1},\ \ldots ,\ v_{p}\bigr \}$ of $p\in {\mathbb {N}}$ vertices $v_{i}$ and a finite edge set $E(G)=\bigl \{e_{1},\ \ldots ,\ e_{q}\bigr \}$ comprising $q\in {\mathbb {N}}$ distinct unordered pairs $e_{\alpha }=\bigl \{v_{i},\ v_{j}\bigr \}$ of distinct vertices such that each element from $V(G)$ appears at least once [13]. The number of vertices $p=|V(G)|$ and edges $q=|E(G)|$ are called the order and size of $G$, respectively. By restricting edges to ensure bond uniqueness (any vertex pair is joined by at most one edge) and forbid loops (each edge must join distinct vertices), molecular structures are naturally represented by such graphs $G$. Each vertex $v_{i}\in V(G)$ has a nonempty neighbor set $S_{i}(G)=\bigl \{v_{j}: \ \{v_{i},\ v_{j}\}\in E(G)\bigr \}$ that lists its adjacent vertices $v_{j}\in V(G)$, and $\mathrm{deg}\bigl (v_{i}\bigr )=\bigl |S_{i}(G)\bigr |$ is called the degree of $v_{i}$. The $p\times p$ square, symmetric and binary vertex adjacency matrix $\mathbf{A}(G)$ of a graph $G$, defined by

$$\begin{aligned} A_{ij}(G) = {\left\{ \begin{array}{ll} 1,\ \ \ &{} \text {if} \ \ \bigl \{v_{i},\ v_{j}\bigr \} \in E(G) \\ 0,\ \ \ &{} \text {otherwise} \end{array}\right. },\ \end{aligned}$$

is in one-to-one correspondence with the molecular structure and is arguably the most natural algebraic representation of topological connectivity. An elementary inductive argument [14] establishes that $\bigl (A^{m}\bigr )_{ij}(G)$ is the total number of $m$ length walks (a sequence of vertices joined consecutively by edges) between vertices $v_{i}$ and $v_{j}$. In particular, the degree of vertex $v_{i}$ corresponding to the valency of atom $i$ in the molecular structure is

$$\begin{aligned} \mathrm{deg}\bigl ( v_{i} \bigr ) = \bigl (A^{2}\bigr )_{ii}(G) = \sum _{j=1}^{p} {\bigl ( A_{ij}(G) \bigr )}^{2} = \sum _{j=1}^{p} A_{ij}(G) = \sum _{\alpha =1}^{q} Z_{i\alpha }(G),\ \end{aligned}$$

so that the $p\times p$ diagonal degree matrix becomes

$$\begin{aligned} \mathbf{D}(G) = \sum _{i=1}^{p} \mathrm{deg}\bigl (v_{i}\bigr ) \mathbf{e}_{i} \, \mathbf{e}_{i}^\mathrm{T} = \mathrm{diag} \Bigl ( \mathrm{deg}\bigl ( v_{1} \bigr ),\ \ldots ,\ \mathrm{deg}\bigl ( v_{p} \bigr ) \Bigr ),\ \end{aligned}$$

where the $\mathbf{e}_{i}$ are natural basis vectors in the coordinate vector space ${\mathbb {R}}^{p}$. Another useful representation of graph connectivity is the $p\times q$ binary vertex-edge incidence matrix $\mathbf{Z}(G)$ defined by

$$\begin{aligned} Z_{i\alpha }(G) = {\left\{ \begin{array}{ll} 1,\ \ \ &{} \text {if} \ \ v_{i} \in e_{\alpha } \\ 0,\ \ \ &{} \text {otherwise} \end{array}\right. }.\ \end{aligned}$$

For each graph $G$ there is an associated line graph $L(G)$ (also called the “derivative” graph [15]) such that $V\bigl (L(G)\bigr )$ is in bijective correspondence with $E(G)$ and

2 $$\begin{aligned} \mathbf{A}\bigl (L(G)\bigr ) = \mathbf{Z}^\mathrm{T}(G) \, \mathbf{Z}(G) - 2 \mathbf{I}_{q} , \end{aligned}$$

where $\mathbf{I}_{q}$ is the $q\times q$ identity matrix. In other words, each edge of $G$ is mapped to a vertex of $L(G)$, while two vertices of the line graph are adjacent if and only if their corresponding edges are incident in $G$ (that is, they share a common endpoint). Furthermore, the associated Kirchhoff matrix $\mathbf{K}(G)=\mathbf{D}(G)-\mathbf{A}(G)$ (also called the Laplacian of $G$) satisfies a similar relationship

$$\begin{aligned} - \mathbf{K}(G) = \mathbf{Z}(G) \, \mathbf{Z}^\mathrm{T}(G) - 2 \mathbf{D}(G) , \end{aligned}$$

and the positive semidefinite *signless* Laplace matrix of $G$ is [16]

$$\begin{aligned} \mathbf{Q}(G) = \mathbf{Z}(G) \, \mathbf{Z}^\mathrm{T}(G) = \mathbf{A}(G) + \mathbf{D}(G). \end{aligned}$$

The spectrum of $\mathbf{K}(G)$ provides a useful consistency check since the algebraic multiplicity of the zero eigenvalue is equal to the number of connected components in $G$ [16]. Hence, for a physically sensible molecular graph $G$, the rank of $\mathbf{K}(G)$ must be $p-1$.

We recall the following definitions and terminology. A cycle graph $C_{r}$ comprises $p=r$ vertices, all of degree $2$, connected in a closed chain by $q=r$ edges. Removing a single edge produces a path graph $P_{r}$ (of order $p=r$ and size $q=r-1$) with two terminal vertices of degree $1$. The complete graph $K_{r}$ on $p=r$ vertices is maximally connected with $q={\textstyle {\frac{1}{2}}}r(r-1)$ edges such that $\bigl \{v_{i},\ v_{j}\bigr \}\in E\bigl (K_{r}\bigr )$ for all distinct $v_{i},\ v_{j}\in V\bigl (K_{r}\bigr )$. A graph $G=\bigl (V(G),\ E(G)\bigr )$ is $k$-partite if the vertices can be partitioned into $k$ disjoint sets, so that $V(G)=\cup _{r=1}^{k}V_{r}(G)$ where $V_{r}(G)\cap V_{s}(G)=\emptyset$ for $r\ne s$. The complete bipartite graph $(k=2)$ is denoted $K_{m,n}$ with $V\bigl (K_{m,n}\bigr )=V_{1}\bigl (K_{m,n}\bigr )\cup V_{2}\bigl (K_{m,n}\bigr )$ and size $p=m+n$ such that $m=\bigl |V_{1}\bigl (K_{m,n}\bigr )\bigr |$ and $n=\bigl |V_{2}\bigl (K_{m,n}\bigr )\bigr |$.

We will also have occasion to consider directed graphs $G=\bigl (V(G),\ A(G)\bigr )$ where edges are replaced by arrows specified by ordered pairs $\bigl (v_{i},\ v_{j}\bigr )\in A(G)$ and oriented with the tail at vertex $v_{i}$ pointing towards the head at vertex $v_{j}$. Associated with each vertex $v_{i}\in V(G)$ are two disjoint neighbor sets $S_{i}^{-}(G)=\bigl \{v_{j}:\,\bigl (v_{i},\ v_{j}\bigr )\in A(G)\bigr \}$ and $S_{i}^{+}(G)=\bigl \{v_{j}\bigl (v_{j},\ v_{i}\bigr )\in A(G)\bigr \}$ where $S_{i}=S_{i}^{-}\cup S_{i}^{+}$. At most one of $S_{i}^{-}$ or $S_{i}^{+}$ may be empty. The indegree of vertex $v_{i}\in V(G)$ is $\mathrm{deg}^{-}\bigl (v_{i}\bigr )=\bigl |S_{i}^{-}(G)\bigr |$ and the outdegree is $\mathrm{deg}^{+}\bigl (v_{i}\bigr )=\bigl |S_{i}^{+}(G)\bigr |$. If $S_{i}^{-}=\emptyset$ so that $\mathrm{deg}^{-}\bigl (v_{i}\bigr )=0$ then vertex $v_{i}$ is called a source and, similarly, if $S_{i}^{+}=\emptyset$ so that $\mathrm{deg}^{+}\bigl (v_{i}\bigr )=0$ then vertex $v_{i}$ is called a sink.

## Intramolecular interactions

**Fig. 1:**
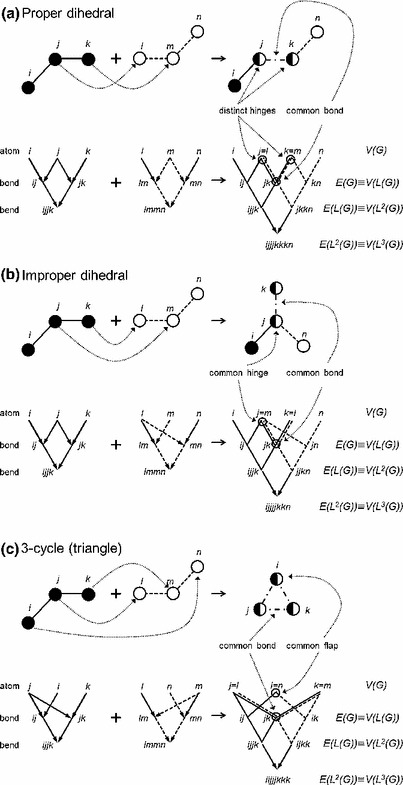
Generic structures of $4$-body interactions represented on the directed acyclic graph $H_{\nu }(G)$ are compared with the physical formation from the combination of two bends with atomic indexes $ijk$ and $lmn$ respectively. **a** Construction of the typical proper dihedral index sequence $ijjjkkkn$ comprising two triplet repeats arising from the distinct hinge atoms $j$ and $k$ that form the shared bond. A similar sequence $iijjjkkk$ arises for the degenerate $3$-cycle structure as shown in (**c**), but where the “flap” atoms are also identified to close the ring. **b** The typical improper dihedral index sequence $ijjjjkkn$ with only a single hinge atom $j$

In a molecular structure, covalently bonded atom pairs are considered to be adjacent. Similarly, a pair of adjacent bonds sharing a common “hinge” atom form a more or less flexible bend. The corresponding $3$-body interaction is often described by an effective potential in terms of the external bond angle $\theta$ supplementary to the angle subtended at the hinge atom [17]. A $4$-body dihedral interaction is associated with a pair of adjacent bends that share a common bond. Two situations are possible [18] (see Fig. 1): “proper” torsions arise when both hinge atoms are distinct, so that one bend is rotated about the other through a dihedral angle $\phi$; while “improper” dihedral interactions link two bends through a common hinge atom, and are defined by a wag angle $\omega$. Proper torsions typically account for geometric restrictions conferred by implicit substituents (usually protons) or lone electron pairs and may be alternatively characterized by a bond lying along the dihedral axis. Conversely, the dihedral axis of an improper torsion does not contain a bond and these interactions are used to constrain planar groups (like rings) or to hinder interconversion of stereocenters.

In graph theoretical terms, this hierarchical organization of interactions is precisely captured by iterated application of the line graph construction inductively defined by

3 $$\begin{aligned} L^{n}(G) = {\left\{ \begin{array}{ll} G\ , \ \ &{} \text {if}\;\quad n = 0 \\ L\bigl (L^{n-1}(G)\bigr )\ , \ \ &{} \text {if}\;\quad n > 0 \end{array}\right. }\ . \end{aligned}$$

A graph $G$ establishes adjacency of vertices (i.e., bonds); the graph $L(G)$ encodes the adjacency of bonds (i.e., bends) in $G$; the graph $L\bigl (L(G)\bigr )=L^{2}(G)$ encodes the adjacency of bends (i.e., dihedrals) in $G$; and so on. Generally, the graph $L^{\nu -2}(G)$ encodes the adjacency of $\nu$-body interactions in $G$. It has been shown [19] that the sequence of graphs $L^{n}(G)$ with $n=0,\ 1,\ 2,\ \ldots$ has only four possible outcomes as $n\rightarrow \infty$:

1. if $G\cong C_{r}$ (a cycle graph on $r$ vertices), then $L^{n}(G)\cong G$ for all $n\in {\mathbb {N}}$ (cycle graphs are the only connected graphs for which $L(G)$ is isomorphic to $G$);
2. if $G\cong K_{1,3}$ (the complete bipartite “claw” graph), then $L^{n}(G)\cong C_{3}$ (a triangle) for all $n\in {\mathbb {N}}$;
3. if $G\cong P_{r}$ (a path graph on $r$ vertices), then $L^{n}(G)\cong P_{\max \{0,r-n\}}$ so each subsequent graph is a shorter path until eventually the sequence terminates at the trivial null graph;
4. otherwise, $G$ is a “prolific” graph [20] so that the sizes of the graphs in the sequence eventually increase without bound,
  $$\begin{aligned} \left| V\bigl (L^{n}(G)\bigr ) \right| \rightarrow \infty \quad \text {as} \quad n \rightarrow \infty .\ \end{aligned}$$

Ghebleh and Khatirinejad [21] have proved an interesting and chemically relevant result concerning the smallest non-negative integer $m$ such that $L^{m}(G)$ is nonplanar: the so-called line index $m=\xi (G)$ of a graph $G$. In particular, if $G$ is not prolific it is easy to see that $L^{n}(G)$ is planar for all $n\ge 0$, but for $G$ prolific then $0\le \xi (G)\le 4$ and a complete characterization of these graphs is possible [21].

### Enumeration

Given a molecular graph $G$, the total number of intramolecular interactions $N_{n}(G)$ involving $n\in {\mathbb {N}}$ connected atoms (vertices on $G$) is generally given by

$$\begin{aligned} N_{n}(G) = \left| V\bigl (L^{n-1}(G)\bigr ) \right| = {\left\{ \begin{array}{ll} \bigl | V(G) \bigr | \ ,&{} \ \ (n = 1) \\ \frac{1}{2} {{\mathrm{Tr}}}{\Bigl ( \mathbf{A}^{2}\bigl ( L^{n-2}(G) \bigr ) \Bigr )} \ ,&{} \ \ (n = 2,\ 3,\ 4,\ \ldots )\ . \end{array}\right. } \end{aligned}$$

Here, $N_{1}(G)$ simply counts the number of $1$-body interactions in the presence of an external field. Elementary combinatorial arguments also establish the handshaking lemma [22]: the number of bonds is just half the total number of incident edges over all vertices so that

$$\begin{aligned} N_\mathrm{bond}(G) = \frac{1}{2} \sum _{v \in V(G)} \mathrm{deg}(v) = {\textstyle {\frac{1}{2}}} {{\mathrm{Tr}}}{\bigl ( \mathbf{D}(G) \bigr )} = {\textstyle {\frac{1}{2}}} \mathbf{u}^\mathrm{T} \mathbf{D}(G) \mathbf{u} = N_{2}(G),\\ \end{aligned}$$

where $\mathbf{u}=\sum _{i=1}^{p}\mathbf{e}_{i}$ is a $p$-vector of ones. By summing the number of possible bond pairs over each vertex, the total bend count is obtained

$$\begin{aligned} \begin{aligned} N_\mathrm{bend}(G)&= \sum _{v \in V(G)} {{\mathrm{deg}(v)} \atopwithdelims (){2}} = \frac{1}{2} \sum _{v \in V(G)} \mathrm{deg}(v) \bigl ( \mathrm{deg}(v) - 1 \bigr ) \\&= {\textstyle {\frac{1}{2}}} {{\mathrm{Tr}}}{\Bigl ( \mathbf{D}(G)\bigl (\mathbf{D}(G) - \mathbf{I}\bigr ) \Bigr )} = {\textstyle {\frac{1}{2}}} {{\mathrm{Tr}}}{\bigl ( \mathbf{D}^{2}(G) \bigr )} - N_{2}(G) \\&= {\textstyle {\frac{1}{2}}} \mathbf{u}^\mathrm{T} \mathbf{D}^{2}(G) \mathbf{u} - N_{2}(G) = N_{3}(G).\\ \end{aligned} \end{aligned}$$

Similarly reckoning the combinations of bond triplets over all vertices yields the number of improper dihedral interactions

$$\begin{aligned} \begin{aligned} N_\mathrm{impr}(G)&= \sum _{v \in V(G)} {{\mathrm{deg}(v)} \atopwithdelims (){3}} = \frac{1}{3!} \sum _{v \in V(G)} \mathrm{deg}(v) \bigl ( \mathrm{deg}(v) - 1 \bigr ) \bigl ( \mathrm{deg}(v) - 2 \bigr ) \\&= {\textstyle {\frac{1}{6}}} \mathbf{u}^\mathrm{T} \mathbf{D}^{3}(G) \mathbf{u} - N_{3}(G) - {\textstyle {\frac{1}{3}}} N_{2}(G).\\ \end{aligned} \end{aligned}$$

Other possible arrangements of three contiguous bonds are just the proper dihedrals and triangular $3$-cycles. In total, these arrangements can be enumerated as follows: for each bond, calculate the product of the number of free edges otherwise incident on each of the two vertices; then sum over all edges to get

$$\begin{aligned} \begin{aligned} N_\mathrm{prop}(G) + 3N_\mathrm{3cyc}(G)&= \sum _{\left\{ v_{i},\ v_{j}\right\} \in E(G)} \Bigl ( \mathrm{deg}\bigl (v_{i}\bigr ) - 1 \Bigr ) \Bigl ( \mathrm{deg}\bigl (v_{j}\bigr ) - 1 \Bigr ) \\&= \frac{1}{2} \sum _{i=1}^{p} \sum _{j=1}^{p} \bigl (D_{ii}(G) - 1\bigr ) A_{ij}(G) \bigl (D_{jj}(G) - 1\bigr ) \\&= {\textstyle {\frac{1}{2}}} \mathbf{u}^\mathrm{T} \mathbf{D}(G) \mathbf{A}(G)\mathbf{D}(G) \mathbf{u} - 2N_{3}(G) - N_{2}(G),\\ \end{aligned} \end{aligned}$$

where we have observed the identity $\sum _{j=1}^{p}A_{ij}(G)=D_{ii}(G)$. The number of $3$-cycles, however, can be obtained directly from the adjacency matrix as

$$\begin{aligned} N_\mathrm{3cyc}(G) = {\textstyle {\frac{1}{3!}}} {{\mathrm{Tr}}}{\bigl ( \mathbf{A}^{3}(G) \bigr )} . \end{aligned}$$

The total $4$-body interaction number $N_{4}$ includes torsions of both types as well as any $3$-cycles present

$$\begin{aligned} N_{4}(G) = N_\mathrm{prop}(G) + 3N_\mathrm{impr}(G) + 3N_\mathrm{3cyc}(G), \end{aligned}$$

but over-counts the improper dihedrals and triangles by distinguishing the three rotational permutations of labels.

Combining these results gives the useful consistency checksums

$$\begin{aligned} N_{1}(G)&= {\textstyle {\frac{1}{2}}} \mathbf{u}^\mathrm{T} \mathbf{A}(G) \mathbf{u}, \\ N_{2}(G)&= {\textstyle {\frac{1}{2}}} \mathbf{u}^\mathrm{T} \mathbf{D}(G) \mathbf{u}, \\ N_{3}(G)&= {\textstyle {\frac{1}{2}}} \mathbf{u}^\mathrm{T} \mathbf{D}^{2}(G) \mathbf{u} - N_{2}(G),\\ N_{4}(G)&= {\textstyle {\frac{1}{2}}} \mathbf{u}^\mathrm{T} \mathbf{D}(G) \mathbf{Q}(G) \mathbf{D}(G) \mathbf{u} - 5N_{3}(G) - 2N_{2}(G), \end{aligned}$$

that involve only the molecular graph $G$.

### Indexing

By definition of the line graph transformation, for $A_{ij}\bigl (L^{n}(G)\bigr )=1$, the indexes $i=\alpha$ and $j=\beta$ point to the edges $e_{\alpha },\ e_{\beta }\in E\bigl (L^{n-1}(G)\bigr )$ that share a common vertex $v_{l}\in V\bigl (L^{n-1}(G)\bigr )$. In turn, these edges $e_{\alpha }=\bigl \{v_{k},\ v_{l}\bigr \}$ and $e_{\beta }=\bigl \{v_{l},\ v_{m}\bigr \}$ are associated with the adjacency matrix of the previous graph in the recursive hierarchy so that $A_{kl}\bigl (L^{n-1}(G)\bigr )=A_{lm}\bigl (L^{n-1}(G)\bigr )=1$. Successively backtracking to the source graph $G$ yields, for each order $n$, a sequence of atomic indexes that participate in an $n$-body interaction. A formal pseudo-code implementation of this procedure is set out in Algorithm 1. Details of each sequence structure completely characterizes the interaction form.
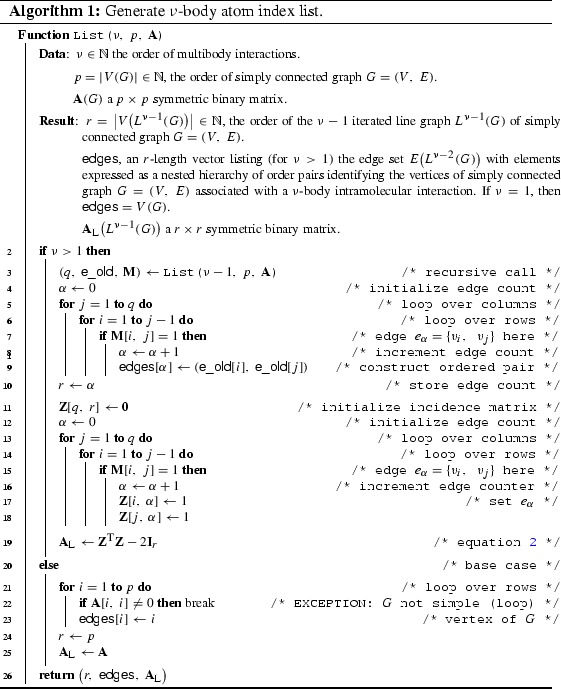


For concreteness, specify a maximum order $\nu$ for the multibody interactions of interest so that $n$-body terms with $n=1,\ 2,\ \ldots ,\ \nu$ are considered. Most commonly in molecular applications $\nu =4$. A convenient representation of the recursive structure defined by (3) is itself a weakly connected $\nu$-partite directed acyclic graph (DAG) denoted $H_{\nu }(G)$ on the union of disjoint vertex sets $\cup _{n=1}^{\nu }V_{n}(G)$ where $V_{n}(G)=V\bigl (L^{n-1}(G)\bigr )$. For clarity, define the vertex set $V\bigl (H_{\nu }(G)\bigr )$ of $H_{\nu }(G)$ with elements $v_{jn}\in V_{n}(G)$. The edge directions induce a partial order relation on the vertices so that $v_{im}\le v_{jn}$ only if $m<n$ where $v_{im}\in V_{m}(G)$ and $v_{jn}\in V_{n}(G)$. Sources of $H_{\nu }(G)$ with indegree $0$ are just the atomic indexes of the molecular graph $G$. All other vertices on $H_{\nu }(G)$ have indegree $2$ as a consequence of the defining property of an edge in $L^{n}(G)$. Similarly, the sinks of $H_{\nu }(G)$ with outdegree $0$ are just the vertices of $L^{\nu -1}(G)$. At each level $n=1,\ 2,\ \ldots ,\ \nu -1$ all other vertices $v_{jn}\in V_{n}(G)$ on $H_{\nu }(G)$ have outdegrees given by $\mathbf{D}\bigl (L^{n-1}(G)\bigr )$. Moreover, the hierarchy of line graphs provides a natural topological order on $H_{\nu }(G)$. Further, the adjacency matrix takes the block tridiagonal form

$$\begin{aligned}&\mathbf{A}\bigl (H_{\nu }(G)\bigr ) \\&\quad = \begin{pmatrix} \mathbf{0} &{}\quad \mathbf{Z}(G) &{}\quad \mathbf{0} &{}\quad \cdots &{}\quad \mathbf{0} &{}\quad \mathbf{0} \\ \mathbf{Z}^\mathrm{T}(G) &{}\quad \mathbf{0} &{}\quad \mathbf{Z}\bigl (L(G)\bigr ) &{}\quad \cdots &{}\quad \mathbf{0} &{}\quad \mathbf{0} \\ \mathbf{0} &{}\quad \mathbf{Z}^\mathrm{T}\bigl (L(G)\bigr ) &{}\quad \mathbf{0} &{}\quad \cdots &{}\quad \mathbf{0} &{}\quad \mathbf{0} \\ \vdots &{}\quad \vdots &{}\quad \vdots &{}\quad \ddots &{}\quad \vdots &{}\quad \vdots \\ \mathbf{0} &{}\quad \mathbf{0} &{}\quad \mathbf{0} &{}\quad \cdots &{}\quad \mathbf{0} &{}\quad \mathbf{Z}\bigl (L^{\nu -2}(G)\bigr ) \\ \mathbf{0} &{}\quad \mathbf{0} &{}\quad \mathbf{0} &{}\quad \cdots &{}\quad \mathbf{Z}^\mathrm{T}\bigl (L^{\nu -2}(G)\bigr ) &{}\quad \mathbf{0} \end{pmatrix}.\\ \end{aligned}$$

By virtue of this DAG structure, each vertex $v_{jn}\in V\bigl (H_{\nu }(G)\bigr )$ is associated with a unique sequence of $2^{n-1}$ atomic indexes $v_{i1}$ that define each $n$-body interaction. For the trivial cases $n=1$ and $n=2$, these sequences correspond respectively with the individual atom list $V(G)$ and the atom pairs $E(G)$ defining the molecular bonds. Bends $(n=3)$ are signified by a pattern of $2^{2}=4$ atoms with a single repeated index identifying the common hinge atom. An $8$-atom sequence classifies a $4$-body interaction and distinguishes between three types as illustrated in Fig. 1. A pair of triply repeated atom indexes identifies the distinct hinge atoms of a proper torsion, provided the remaining two indexes are different (Fig. 1a). Otherwise, a common pair of “flap” atoms signals a degenerate $3$-cycle where only three atom indexes appear (Fig. 1c). Each triangle generates ${}^{3}C_{2}=3$ such sequences by the arbitrary choice of a single common bond. The single common hinge of an improper dihedral corresponds to a fourfold repeated index among four distinct labels (Fig. 1b). Again, three sequences are generated for each improper dihedral by arbitrary assignment of the shared bond.

## Examples

### A toy model: methylcyclopropane

**Fig. 2:**
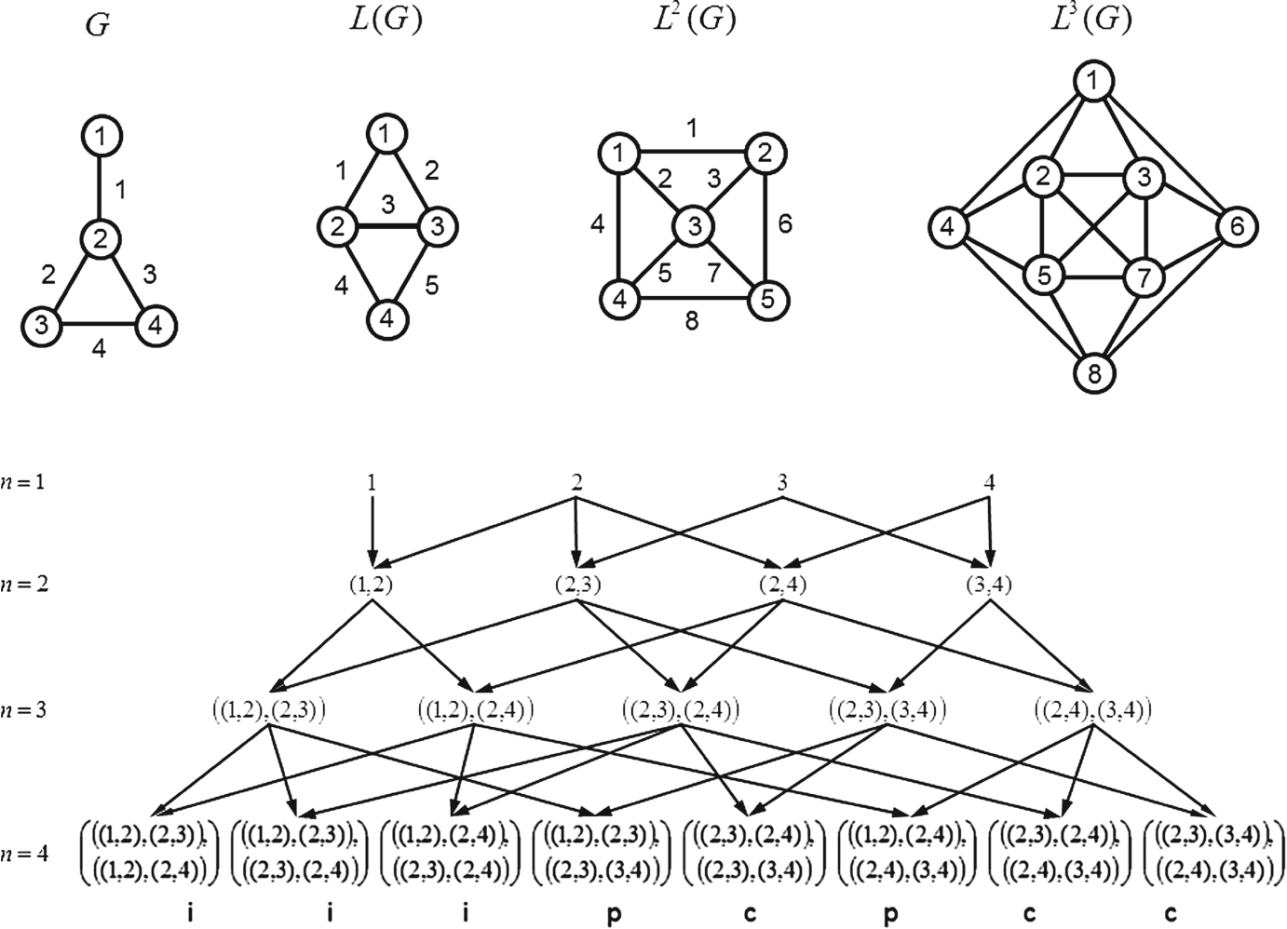
A plausible effective model of methylcyclopropane is represented by the graph $G$. The iterated line graphs $L^{n-1}(G)$ are also shown for $n=2,\ 3,\ 4$. Vertices $v_{im}\in V_{m}(G)=V\bigl (L^{m-1}(G)\bigr )$ and edges $e_{km}=\bigl \{v_{im},\ v_{jm}\bigr \}$ are labelled. The corresponding directed acyclic graph (DAG) generated by the line graph hierarchy is given where the vertices at each level $n$ are denoted by the inherited sequence of atomic labels from $G$ as indicated by the directed edges. Inspection of $G$ confirms the $4$-body interactions identified by the DAG sinks and comprise of: two proper torsions (denoted “p”) with distinct hinge atoms $2$-$3$ and $2$-$4$, respectively; a single improper dihedral (denoted “i”) with common hinge $2$; and a single $3$-cycle (denoted “c”) of atoms $2$, $3$ and $4$

The molecular graph $G$ indicated in Fig. 2 presents amongst other possibilities a plausible lumped model for methylcyclopropane where hydrogen atoms are absorbed onto the carbon backbone in the usual way. Direct inspection of $G$ immediately establishes four $1$-body interactions in the presence of an external field (that is, the number of “atoms” $N_{1}=4$) and also four $2$-body bonds $\bigl (N_{2}=4\bigr )$. Atom $2$ is the hinge for three distinct $3$-body bends with a further two hinged at atoms $3$ and $4$ respectively, to give $N_{3}=5$. Among the $4$-body interactions there are two proper torsions $\bigl (N_\mathrm{prop}=2\bigr )$ with distinct hinge atoms $2$-$3$ and $2$-$4$, respectively, as well as a single improper dihedral $\bigl (N_\mathrm{impr}=1\bigr )$ with common hinge atom $2$. A single $3$-cycle is present $\bigl (N_\mathrm{3cyc}=1\bigr )$ so that $N_{4}=2+3\times (1+1)=8$. Adjacency matrices for the iterated line graphs necessary for describing interactions up to the $4$-body level are given by

$$\begin{aligned}&\mathbf{A}(G) = \begin{pmatrix} 0 &{}\quad 1^{1} &{}\quad 0 &{}\quad 0 \\ 1^{1} &{}\quad 0 &{}\quad 1^{2} &{}\quad 1^{3} \\ 0 &{}\quad 1^{2} &{}\quad 0 &{}\quad 1^{4} \\ 0 &{}\quad 1^{3} &{}\quad 1^{4} &{}\quad 0 \end{pmatrix} , \quad \mathbf{A}\bigl (L(G)\bigr ) = \begin{pmatrix} 0 &{}\quad 1^{1} &{}\quad 1^{2} &{}\quad 0 \\ 1^{1} &{}\quad 0 &{}\quad 1^{3} &{}\quad 1^{4} \\ 1^{2} &{}\quad 1^{3} &{}\quad 0 &{}\quad 1^{5} \\ 0 &{}\quad 1^{4} &{}\quad 1^{5} &{}\quad 0 \end{pmatrix} ,\\&\mathbf{A}\bigl (L^{2}(G)\bigr ) = \begin{pmatrix} 0 &{}\quad 1^{1} &{}\quad 1^{2} &{}\quad 1^{4} &{}\quad 0 \\ 1^{1} &{}\quad 0 &{}\quad 1^{3} &{}\quad 0 &{}\quad 1^{6} \\ 1^{2} &{}\quad 1^{3} &{}\quad 0 &{}\quad 1^{5} &{}\quad 1^{7} \\ 1^{4} &{}\quad 0 &{}\quad 1^{5} &{}\quad 0 &{}\quad 1^{8} \\ 0 &{}\quad 1^{6} &{}\quad 1^{7} &{}\quad 1^{8} &{}\quad 0 \end{pmatrix}.\\ \end{aligned}$$

For added clarity, the edge index associated with each adjacent vertex pair is indicated by the superscript. From these matrices, the DAG obtained that represents the line graph hierarchy is shown in Fig. 2. The atomic index sequences automatically generated on the DAG, particularly at the $4$-body level $(n=4)$, confirm the informal analysis of the molecular graph. It is easy to show that the complete graph on five vertices $K_{5}$ is a minor of $L^{3}(G)$, whence it follows from the theorem of Wagner [23] that $L^{3}(G)$ is nonplanar. All of $G$, $L(G)$ and $L^{2}(G)$ are manifestly planar (see Fig. 2) so the line index $\xi (G)=3$ in accord with the result of Ghebleh and Khatirinejad [21].

### Bile salt: taurocholate

**Fig. 3:**
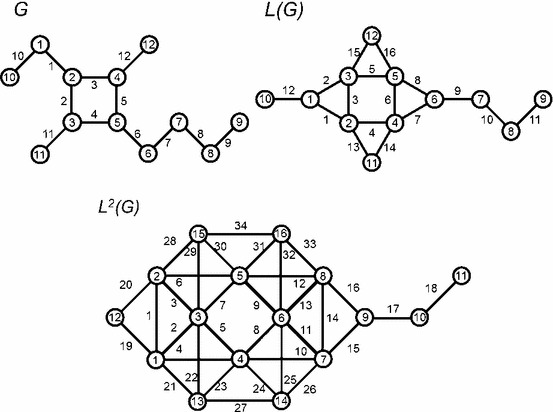
Effective coarse grained bile salt model of Vila Verde and Frenkel [24]. The molecular graph $G$ is shown along with the iterated line graphs $L(G)$ and $L^{2}(G)$. Vertices and edges are labelled

**Fig. 4:**
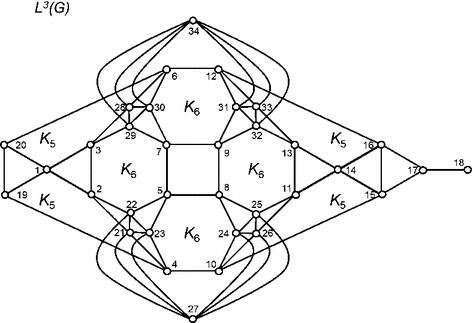
Effective coarse grained bile salt model of Vila Verde and Frenkel [24]. The molecular line graph $L^{3}(G)$. To simplify the diagram, subgraphs corresponding to complete graphs $K_{q}$ on $q$ vertices ( $q$-cliques of $L^{3}(G)$) have been rendered as pentagons $(q=5)$ and hexagons $(q=6)$

A chemically relevant example, central to the hydrolysis and solubilisation of lipid associated with food digestion in the human lower gastrointestinal tract, are the bile salt and bile acid surfactants. Vila Verde and Frenkel [24] have proposed an effective coarse-grained model of trihydroxy bile salts (taurocholate) for a molecular dynamics study of micelle formation that is related to the rate and extent of nutrient absorption by intestinal cells. The authors proposed a “three-to-one” mapping scheme, that groups three carbon or nitrogen atoms into a single bead, to arrive at the molecular graph $G$ shown in Fig. 3. Iterated line graphs up to $L^{3}(G)$ are collected in Figs. 3 and 4. Table 1 lists the vertices for the corresponding DAG description of the hierarchy as sequences of bead indexes.

**Table 1:** Vertex sequences associated with the directed acyclic graph (DAG) description of the line graph hierarchy for the effective bile salt model of Vila Verde and Frenkel [24]

$N_{n}$	$n=1$	$n=2$	$n=3$	$n=4$	
1	1	(1, 2)	((1, 2), (2, 3))	(((1, 2), (2, 3)), ((1, 2), (2, 4)))	i
2	2	(2, 3)	((1, 2), (2, 4))	(((1, 2), (2, 3)), ((2, 3), (2, 4)))	i
3	3	(2, 4)	((2, 3), (2, 4))	(((1, 2), (2, 4)), ((2, 3), (2, 4)))	i
4	4	(3, 5)	((2, 3), (3, 5))	(((1, 2), (2, 3)), ((2, 3), (3, 5)))	p
5	5	(4, 5)	((2, 4), (4, 5))	(((2, 3), (2, 4)), ((2, 3), (3, 5)))	p
6	6	(5, 6)	((3, 5), (4, 5))	(((1, 2), (2, 4)), ((2, 4), (4, 5)))	p
7	7	(6, 7)	((3, 5), (5, 6))	(((2, 3), (2, 4)), ((2, 4), (4, 5)))	p
8	8	(7, 8)	((4, 5), (5, 6))	(((2, 3), (3, 5)), ((3, 5), (4, 5)))	p
9	9	(8, 9)	((5, 6), (6, 7))	(((2, 4), (4, 5)), ((3, 5), (4, 5)))	p
10	10	(1, 10)	((6, 7), (7, 8))	(((2, 3), (3, 5)), ((3, 5), (5, 6)))	p
11	11	(3, 11)	((7, 8), (8, 9))	(((3, 5), (4, 5)), ((3, 5), (5, 6)))	i
12	12	(4, 12)	((1, 2), (1, 10))	(((2, 4), (4, 5)), ((4, 5), (5, 6)))	p
13			((2, 3), (3, 11))	(((3, 5), (4, 5)), ((4, 5), (5, 6)))	i
14			((3, 5), (3, 11))	(((3, 5), (5, 6)), ((4, 5), (5, 6)))	i
15			((2, 4), (4, 12))	(((3, 5), (5, 6)), ((5, 6), (6, 7)))	p
16			((4, 5), (4, 12))	(((4, 5), (5, 6)), ((5, 6), (6, 7)))	p
17				(((5, 6), (6, 7)), ((6, 7), (7, 8)))	p
18				(((6, 7), (7, 8)), ((7, 8), (8, 9)))	p
19				(((1, 2), (2, 3)), ((1, 2), (1, 10)))	p
20				(((1, 2), (2, 4)), ((1, 2), (1, 10)))	p
21				(((1, 2), (2, 3)), ((2, 3), (3, 11)))	p
22				(((2, 3), (2, 4)), ((2, 3), (3, 11)))	p
23				(((2, 3), (3, 5)), ((2, 3), (3, 11)))	i
24				(((2, 3), (3, 5)), ((3, 5), (3, 11)))	i
25				(((3, 5), (4, 5)), ((3, 5), (3, 11)))	p
26				(((3, 5), (5, 6)), ((3, 5), (3, 11)))	p
27				(((2, 3), (3, 11)), ((3, 5), (3, 11)))	i
28				(((1, 2), (2, 4)), ((2, 4), (4, 12)))	p
29				(((2, 3), (2, 4)), ((2, 4), (4, 12)))	p
30				(((2, 4), (4, 5)), ((2, 4), (4, 12)))	i
31				(((2, 4), (4, 5)), ((4, 5), (4, 12)))	i
32				(((3, 5), (4, 5)), ((4, 5), (4, 12)))	p
33				(((4, 5), (5, 6)), ((4, 5), (4, 12)))	p
34				(((2, 4), (4, 12)), ((4, 5), (4, 12)))	i

At each level $n$, the inherited sequence of atomic labels from the molecular graph $G$ is listed. Inspection of $G$ confirms the $4$-body interactions identified by the DAG sinks and comprise of: $22$ proper torsions (denoted “p”) and $4$ improper dihedrals (denoted “i”) with no $3$-cycles

Overall, the counts of bonds, bends, proper torsions and improper dihedrals are obtained as follows,

$$\begin{aligned} N_\mathrm{bond} = 12,\ \quad N_\mathrm{bend} = 16,\ \quad N_\mathrm{prop} = 22,\ \quad N_\mathrm{impr} = 4,\ \end{aligned}$$

where $N_{4}=22+3\times 4=34$. Clearly, there are no $3$-cycles in this example so $N_\mathrm{cyc}=0$. It is easy to verify that $L^{2}(G)$ admits the minor $K_{5}$ and hence, by Wagner’s theorem [23], it follows that $L^{2}(G)$ is nonplanar. Both $G$ and $L(G)$ are manifestly planar so the line index $\xi (G)=2$ in accord with the result of Ghebleh and Khatirinejad [21].

## Conclusion

The line graph transformation provides a practical and elegant theoretical tool for exhaustively enumerating and indexing many-body intramolecular interactions. Given a suitable graphical representation of a molecular structure, an explicit pseudo-code implementation of the recursive line graph algorithm is given for automatically generating complete canonical lists of atomic indexes associated with each interaction order. No attempt has been made to computationally optimize this algorithm or the associated data structures. Instead, clarity of exposition is the main objective here. We anticipate the main application will involve embedding the algorithm within a Monte Carlo or Molecular Dynamics simulation code where other implementation details will determine the most efficient realization. In accord with common practice, intramolecular interactions up to order $4$ have been considered (bonds, bends and dihedrals), but the method can be extended to arbitrarily many atomic centers. Higher order interactions will involve increasingly many sub-type variations and polycyclic structures. Two specific examples are discussed: a toy model of methylcyclopropane and a published effective potential model of taurocholate bile salt [24] that is relevant for the study of digestive processes in the human lower gastrointestinal tract.
